# Development process and initial validation of the Ethical Conflict in Nursing Questionnaire-Critical Care Version

**DOI:** 10.1186/1472-6939-14-22

**Published:** 2013-06-01

**Authors:** Anna Falcó-Pegueroles, Teresa Lluch-Canut, Joan Guàrdia-Olmos

**Affiliations:** 1 Department of Fundamental Care and Medical-Surgical Nursing, Campus of Health Science of Bellvitge, Nursing School, University of Barcelona, Central Pavilion, 3r floor, L’08907, Hospitalet de Llobregat, Barcelona, Spain; 2 Department of Public Health, Mental Health and Midwife Nursing, Health Science Campus of Bellvitge, Nursing School, University of Barcelona, Barcelona, Spain; 3 Methodology of Behavioural Sciences, Faculty of Psychology, Research Institute on Brain, Cognition and Behaviour (IR3C), University of Barcelona, Barcelona, Spain

## Abstract

**Background:**

Ethical conflicts are arising as a result of the growing complexity of clinical care, coupled with technological advances. Most studies that have developed instruments for measuring ethical conflict base their measures on the variables ‘frequency’ and ‘degree of conflict’. In our view, however, these variables are insufficient for explaining the root of ethical conflicts. Consequently, the present study formulates a conceptual model that also includes the variable ‘exposure to conflict’, as well as considering six ‘types of ethical conflict’. An instrument was then designed to measure the ethical conflicts experienced by nurses who work with critical care patients. The paper describes the development process and validation of this instrument, the Ethical Conflict in Nursing Questionnaire Critical Care Version (ECNQ-CCV).

**Methods:**

The sample comprised 205 nursing professionals from the critical care units of two hospitals in Barcelona (Spain). The ECNQ-CCV presents 19 nursing scenarios with the potential to produce ethical conflict in the critical care setting. Exposure to ethical conflict was assessed by means of the Index of Exposure to Ethical Conflict (IEEC), a specific index developed to provide a reference value for each respondent by combining the intensity and frequency of occurrence of each scenario featured in the ECNQ-CCV. Following content validity, construct validity was assessed by means of Exploratory Factor Analysis (EFA), while Cronbach’s alpha was used to evaluate the instrument’s reliability. All analyses were performed using the statistical software PASW v19.

**Results:**

Cronbach’s alpha for the ECNQ-CCV as a whole was 0.882, which is higher than the values reported for certain other related instruments. The EFA suggested a unidimensional structure, with one component accounting for 33.41% of the explained variance.

**Conclusions:**

The ECNQ-CCV is shown to a valid and reliable instrument for use in critical care units*.* Its structure is such that the four variables on which our model of ethical conflict is based may be studied separately or in combination. The critical care nurses in this sample present moderate levels of exposure to ethical conflict. This study represents the first evaluation of the ECNQ-CCV.

## Background

Ethical conflicts have been analysed for several years in various clinical contexts. Research suggests that such conflicts are on the rise in the nursing field, due both to the increasing complexity of care and the scientific and technological advances which have been made in recent decades. In this regard, critical care units are a setting that is especially prone to conflict [1-14]. Various authors have suggested that the ethical conflicts experienced by critical care nurses stem from three main sources: the relationships with patients and their families, the provision of certain treatments and/or the characteristics of the setting in which the clinical team works. As regards the first of these, the decision-making process comes up against issues such as the difficulty of ensuring informed consent, a failure to respect confidentiality or the lack of protection of the patient’s interests [5,9,15-21]. With respect to the provision of certain treatments, nurses may experience conflict when asked to administer treatment they regard as overly aggressive, when pain management seems to be deficient or when it becomes necessary to limit the use of life support procedures [1,5,15-17,22-24]. Finally, in relation to workplace dynamics, conflict may arise if nurses are not fully involved in the decision-making process or if they feel the work environment makes it difficult to consider questions of a bioethical nature [4,12,15,17,19-25]. Such situations have the potential to produce different ethical conflicts in the individual, and these conflicts will reflect the root of the difficulty in making the right decision. In this context, Andrew Jameton [26] coined the term *moral distress* and identified three types of ethical conflict which nurses may experience in the clinical setting: *moral uncertainty*, *moral dilemma* and *moral distress.* In a situation of moral uncertainty the professional is either unsure whether there is an ethical problem or not, or recognizes that there is such a problem but is unclear about the ethical principles involved. Moral dilemmas arise when the professional must choose between two or more morally correct principles, each of which would lead to a distinct course of action. Finally, moral distress is felt when the professional recognizes the ethical principles involved and knows the right thing to do but is constrained by something or somebody from acting accordingly. Some years after Jameton’s work was first published Judith Wilkinson [15] referred to what she called *moral outrage*, a type of ethical conflict in which the professional experiences a sense of impotence in the face of an immoral action performed by others. In 1993 Jameton included this type of conflict in his own classification [27].

Although a considerable body of research has considered the types of ethical conflict experienced by nurses, only a few studies have developed instruments for measuring these conflicts. The principal instruments to date have focused on the evaluation of moral distress and stress of conscience. The *Moral Distress Scale*, developed by Corley [1], was the first instrument of its kind and was subsequently adapted by various authors [6,10,11,20,21,28-30]. Kälvemark-Sporrong’s *Moral Distress Questionnaire*[31] was designed to explore the relationship between moral distress, ethical competence and the ability to tolerate stress among health professionals. This instrument was also adapted in subsequent research [25,31-33]. Finally, the *Stress of Conscience Questionnaire* was developed by Glasberg [34] to examine the relationship between stress of conscience and burnout, a relationship that was later confirmed by other studies [35-37].

**Figure 1:**
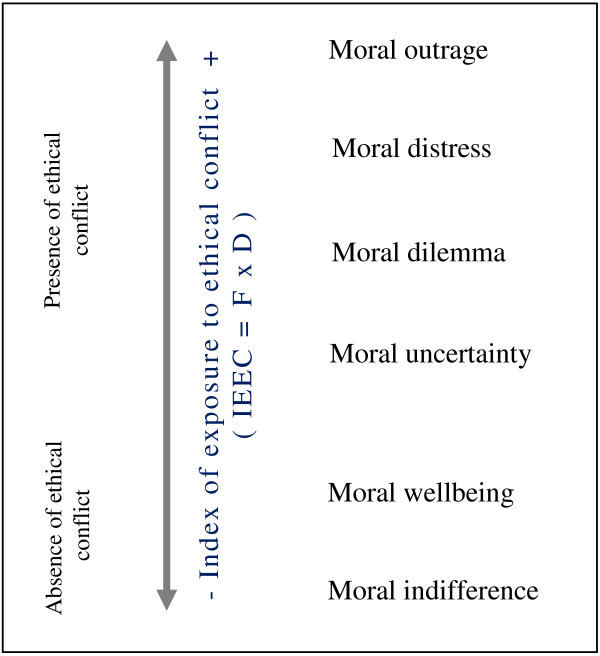
Model for the analysis of ethical conflict.

Most of the research which has set out to analyse ethical conflicts has focused predominantly on two variables: the frequency with which situations of conflict arise and the degree of ethical conflict perceived by the individual concerned. As regards the relationship between these two variables, Corley [23] and Pauly [29] report there to be a positive relationship (p < 0.01) in the context of moral distress. Similarly, Glasberg’s construct *stress of conscience*[34,35] was described as the product of these two variables. In our view, however, the sole use of these two variables (i.e. frequency and degree of ethical conflict) to analyse ethical conflicts is insufficient when it comes to explaining the root of the conflict or the difficulty of making the correct decision from a moral perspective. Indeed, it is noteworthy that research has yet to consider as a whole the different types of ethical conflict described by Jameton [27] and Wilkinson [15]. Neither have we found any studies that take into account the absence of moral conflict as a positive perspective inside a model. Consequently, we have formulated a model for the analysis of ethical conflict (Figure 1) that is based on the following premises: a) in order to study ethical conflict it is necessary to consider four variables: frequency of conflict, degree of conflict, exposure to conflict and type of conflict; b) the variable ‘exposure to conflict’ is the product of the variables ‘frequency’ and ‘degree of conflict’; c) the variable ‘type of conflict’ should take into account the continuum between the presence and absence of ethical conflict. The presence of ethical conflict would correspond to the four categories or types of conflict described by Jameton [26,27] and Wilkinson [15]: *moral uncertainty*, *moral dilemma, moral distress* and *moral outrage.* In order to capture the absence of ethical conflict our model defines a further two categories: moral wellbeing and moral indifference [24]. Moral wellbeing refers to a positive state in which moral thought and action are clearly coherent with one another. Moral indifference describes the stance of an individual who neither shows interest in nor takes a position on a matter of ethical concern.

Based on this model of ethical conflict we then developed an instrument for evaluating its components. The focus of the present study is on critical care nursing, and the paper describes the development and validation of the instrument designed to measure ethical conflicts in this setting, the Ethical Conflict in Nursing Questionnaire-Critical Care Version (ECNQ-CCV).

## Methods

### Item generation

The initial pool of items was created through an extensive review of the literature on sources of ethical conflict in nursing and in the critical care setting. During the search particular emphasis was placed on identifying and selecting care scenarios that generated an ethical problem, a moral dilemma or moral distress*.* A total of 31 situations involving an ethical conflict were identified. Those situations which produced conflict of another kind, for example, staff-related or financial, were excluded.

The 31 scenarios were then analysed and adapted to the specific context of critical care units, grouping them into 11 areas with the potential to produce an ethical conflict. These areas were as follows: informed consent, confidentiality, withholding and withdrawing treatments, the patient’s interests, characteristics of an ethical environment, procedures and treatments, interprofessional relationships, moral agency and professional values, privacy, research tasks and resource management. In order to reduce the number of scenarios we then grouped together any that were related to one another, the result being a total of 19 situations, each of which was represented by one item on the questionnaire. The 19 scenarios were then analysed in accordance with the Code of Ethics of the International Council of Nurses [38] in order to identify which articles of the Code might be implicated in each case.

### The questionnaire: content validity and pilot study

The instrument, which we called the Ethical Conflict in Nursing Questionnaire-Critical Care Version (ECNQ-CCV), was developed in Spanish and initially comprised the 19 care scenarios with the potential to produce an ethical conflict in nurses working in critical care units.

In order to test the content validity of this initial version of the ECNQ-CCV each of its items was rated by two committees of experts. One committee (the Ethics Committee) comprised four experts in the field of ethics and nursing ethics, while the other was formed by four experts in critical care nursing (the Critical Care Committee). Each member of both committees evaluated the ECNQ-CCV according to the following two parameters: ‘Relevance of the item for exploring ethical conflict’ (RI) and ‘Degree of ethical conflict’ (DEC). The members of the Ethics Committee also evaluated the items according to the degree of agreement in the ‘Definition of ethical concepts’ (DEFEC), whereas the Critical Care Committee was asked to consider, as a third parameter, the ‘Frequency of occurrence of each scenario’ (FO). Ratings of the four parameters (RI, DEC, DEFEC and FO) were made using a Likert scale with five or six response options, and there was also a section in which the experts could make any comments or suggestions they felt were necessary.

Content validity was then assessed by means of two procedures. The data corresponding to the parameters RI, DEC and FO were analysed according to the consensus method described by Fehring [39]. This method classifies the degree of agreement into four categories: 0 to 0.24 = no consensus, 0.25 to 0.49 = little consensus, 0.50 to 0.74 = considerable consensus, and 0.75 or higher = strong consensus. The results showed that three items (Item 2, Item 13 and Item 16) yielded values below 0.50 with respect to RI and DEC, thereby suggesting that they should be eliminated. However, one of these items (Item 2) produced a value above 0.50 on FO and it was therefore retained. The other two items were replaced with two new items suggested by some of the experts. One item referred to the effect of a lack of resources on care, while the other concerned institutional interests coming before those of the patient.

The parameter DEFEC was analysed by using Kendall’s coefficient of concordance to determine the degree of agreement between experts. The degree of agreement obtained was sufficient: W = 0.519 for the four types of ethical conflict and W = 0.750 for the degree of agreement between the categories *moral dilemma* and *moral distress*.

The questionnaire therefore comprised 19 items, each of which described a critical care scenario in which nurses might experience an ethical conflict. For each item, three questions were formulated, corresponding to the parameters ‘frequency of occurrence of the ethical conflict’ , ‘degree of perceived ethical conflict’ and ‘type of ethical conflict experienced’ (Additional file 1). The variable ‘frequency of occurrence’ was rated according to six categories: never, almost never, at least once a year, at least once every six months, at least once a month, and at least once a week. ‘Degree of conflict’ was rated across five categories: no problem at all, mildly problematic, fairly problematic, considerably problematic, and highly problematic. Finally, six categories were used to rate the ‘type of ethical conflict experienced’. Four of these were the four types of ethical conflict defined by Jameton [26,27] and Wilkinson [15]: moral uncertainty, moral dilemma, moral distress and moral outrage*.* The remaining two categories referred to moral states in which no ethical conflict was present: moral wellbeing and moral indifference [24].

The instrument in this form was then piloted with a sample of 20 nurses from the critical care unit of a hospital in the city of Barcelona, the characteristics of which were similar to those of the two hospitals chosen for the main study. In this pilot study, 72% of the nurses surveyed considered that the ECNQ-CCV had sufficient scope and 28% regarded it as acceptable. 78% affirmed it was sufficiently clear and the remaining 22% declared it was clear. These results meant that no substantial changes needed to be made to the design or content of the questionnaire.

### Index of Exposure to Ethical Conflict (IEEC)

As mentioned earlier the model of ethical conflict on which this study is based includes the variable ‘exposure to conflict’. This variable results from the relationship between the frequency with which an individual encounters an ethical conflict and the degree of conflict that such situations produce in that individual. This relationship is best regarded as a continuum. At one extreme there would be no ethical conflict: the individual has either never been in a situation of potential conflict, or has been but no conflict was produced (zero frequency – zero intensity). The other end of the continuum would be anchored by maximum or strong ethical conflict: the individual frequently encounters situations of ethical conflict and the degree of conflict produced is very intense (high frequency – high intensity). Between these two extremes would lie intermediate combinations: low frequency – high intensity; high intensity - low frequency.

In order to estimate ‘exposure to ethical conflict’ we developed a specific index, the Index of Exposure to Ethical Conflict (IEEC), which yields a reference value for each subject. The IEEC is calculated by means of the following equation:

$$\text{IEEC}=\sum_{i=1}^{19}\left(F_{i}\times D_{i}\right)$$

where (*F*_*i*_) is the frequency of occurrence of each situation (*Item*_*i*_) and (*D*_*i*_) is the degree of intensity corresponding to that situation. The value of the IEEC therefore provides an estimate of the frequency and intensity with which a given subject experiences each of the 19 scenarios listed within the ECNQ-CCV, the sum of its products reflecting the interaction between the ratings given for frequency and intensity. Thus, the IEEC enables respondents to be evaluated and ordered according to the degree of ethical conflict they experience. The possible score for each item ranges from 0 to 25: 0 is the product of the categories Zero Frequency – Zero Intensity (‘0 - Never’ × ‘0 - No problem at all’) and 25 is the product of the categories High Frequency – High Intensity (‘5 – At least once a week’ × ‘5 – Highly problematic’). The range of the IEEC is therefore 0 to 475, the upper limit being the product of 19 × 25 (19 = the number of scenarios included in the ECNQ-CCV with the potential to produce ethical conflict × 25 = maximum value of the IEEC for each situation).

### Participants and setting

Participants were critical care nurses with at least one year of professional experience who were recruited through two tertiary level hospitals, one in Barcelona (Spain) and the other in the metropolitan area of the same city. Both centres were linked to the University of Barcelona. The sample comprised 205 nurses who worked in the critical care units of these two hospitals. As the total number of critical care nurses at these hospitals was 292, the sample accounted for 70% of the population.

Socio-demographic, academic, professional and institutional information regarding the sample was collected by means of a data sheet comprising 20 questions. This data sheet was attached to the ECNQ-CCV.

Administration of both the ECNQ-CCV and the complementary data sheet took place during October and November 2009.

### Statistical analysis

All data were analysed using PASW v19 for Windows. The tests of normality and the analyses of the reliability and construct validity of the Ethical Conflict Nursing Questionnaire were based on the IEEC. Internal consistency was calculated using Cronbach’s α coefficient. Construct validity was assessed by means of Exploratory Factor Analysis (EFA), examining the scree plot in order to interpret dimensionality. Measures of central tendency were studied for the IEEC, and the Kolmogorov-Smirnov and Shapiro Wilks tests were applied in order to assess the goodness of fit of the distributions. Variances were also analyzed with ANOVA test or the corresponding non-parametric test in order to compare the means of the groups and see if there was an influence between IEEC and the variable type of conflict.

### Ethical considerations

The study was approved by the Clinical Research Ethics Committee of Ciutat Sanitària Bellvitge and the Clinical Research Ethics Committee of Hospital Clínic Universitari de Barcelona, as well as by the respective nursing management teams. Participants were informed about the authorship and purpose of the research, and were told that all data would remain anonymous and confidential.

## Results and discussion

The Ethical Conflict Nursing Questionnaire, ECNQ-CCV, was easy to administer and required only 15 minutes to complete. The wording of items was regarded as clear.

Most of the sample were female (83.7%) and the average age (based on the 171 nurses who provided this information) was 38.8 years (SD 10.6, range 23–61). The majority of nurses were involved in patient care (94.6%), were experienced (31.5% had more than 20 years of experience) and had postgraduate training in caring for critically ill patients (69%). Table 1 shows the descriptive data for the sample.

**Table 1:** Sample demographic characteristics

**Variables/categories**	**n (%)**	**Mean ± SD**
		**(Md/age range)**
Age (For a n = 170)		38.8 ± 10.687
		(36/23–61)
Sex		
Female	170 (83.7	
Male	30 (8.14)	
Hospital		
Hospital 1 (Barcelona’s metropolitan area)	100 (49.3)	
Hospital 2 (Barcelona city)	103 (50.7)	
Workplace		
H1 1–1 CCU - *Respiratory diseases *principally	41 (2,20)	
H1 2–1 CCU - *NeuroTrauma diseases principally*	13 (4.6)	
H1 2–2 CCU - *Wearing and semi-critical patients*	18 (8.9)	
H1 3–2 CCU - *Cardiac surgery*	25 (3.12)	
H2 - CCU *Respiratory diseases*	15 (4.7)	
H2 - CCU *Surgery Unit*	21 (3.10)	
VH2 - CCU *Digestive and Hepatic diseases*	17 (4.8)	
H2 - CCU Coronary	16 (7.9)	
H2 - CCU Cardiac surgery	15 (4.7)	
H2- CCU Intensive vigilance	19 (4.9)	
*Dnk/Na*	3 (1.4)	
Role		
General nurse	192 (94.6)	
Clinical supervisor	8 (3.9)	
Head nurse	2 (1)	
*Dnk/Na*	1 (0.5)	
Shift		
Days only	62 (5.30)	
Evenings	57 (1.28)	
Days weekend	11 (4.5)	
Evenings weekend	6 (3)	
Nights (Monday shift and Tuesday shift)	64 (5.31)	
*Prefer not to answer*	3 (1.5)	
Years as a nurse		
1 - 5	38 (7.18)	
5 - 10	36 (7.17)	
10 - 15	31 (3.15)	
15 - 20	32 (8.15)	
> 20	64 (5.31)	
*Prefer not to answer*	2 (1)	
Years in critical care units		
1 - 5	50 (6.24)	
5 - 10	41 (2.20)	
10 - 15	31 (3.15)	
15 - 20	38 (7.18)	
> 20	39 (2.19)	
*Prefer not to answer*	4 (2)	
Postgraduate training in critical care units		
Yes	140 (69)	
No	60 (5.29)	
*Prefer not to answer*	3 (1.5)	

Definition of abbreviation: CCU, Critical Care Unit; H1, Hospital 1; H2, Hospital 2.

The analyses of reliability and validity and the tests of normality were based on a sub-sample of 164 nurses, corresponding to those who completed the whole of the ECNQ-CCV.

### Reliability of the ECNQ-CCV

The overall α value for the ECNQ-CCV was 0.882, while the α value if the item is eliminated ranged between 0.871 and 0.881. These results suggest that none of the 19 items should be eliminated. Indeed, removal of any one item led to only a minimal improvement in the reliability coefficient. The overall value of α = 0.882 demonstrates high reliability according to the criterion of Graham and Lilly [40]. Comparison of these values with the α coefficients obtained for other instruments designed to measure similar constructs shows that the overall α value for the ECNQ-CCV is higher than that reported for both the *Moral Distress Questionnaire* (α = 0.78) [25,31] and the *Stress of Conscience Questionnaire* (α = 0.83) [35,36].

### Validity of the ECNQ-CCV

The Kaiser-Meyer-Olkin index (0.871) and Bartlett’s test of sphericity (χ^2^ = 998.505, with df = 171 and p < 0.001) confirmed the suitability of reducing the dimensionality of the ECNQ-CCV. Exploratory Factor Analysis (EFA) revealed a principal component that explained 33.41% of the variance. This result, together with the scree plot, supports a unidimensional structure for the ECNQ-CCV. This unidimensionality can be attributed to the global nature of the concept ‘ethical conflict’ which the instrument evaluates. The EFA also showed that all the items had loadings above 0.40 (p < .001), thereby indicating that each of them is relevant to the measurement of the study phenomenon (Table 2). The unidimensional structure of the ECNQ-CCV distinguishes it from other instruments designed to evaluate similar constructs, and which have two [31,34], three [22,23,25] or four dimensions [41] . To validate the factor structure estimated from the EFA were obtained fit indices of the same structure from Confirmatory Factor Analysis models (CFA) to establish more security goodness of the factor structure. Thus, by robust and elliptical estimation technique, due to sample size and some difficulty with the multinormality condition of observed variables, was obtained adjusted fit index that allow the aforementioned structure was assumed (χ^2^ = 243.45; p = 0.189; Comparative Fit Index CFI = 0.972; Standardized Root Mean Residual Square SRMRS = 0.004).

**Table 2:** Results of the Exploratory Factor Analysis (EFA)

	**Components**			
	**Component 1**	**Component 2**	**Component 3**	**Component 4**
Item 11	0.737			
Item 17	0.655	-0.515		
Item 1	0.643			
Item 2	0.629			
Item 8	0.625			
Item 9	0.623			
Item 14	0.616			
Item 6	0.601			
Item 3	0.582			
Item 15	0.582			
Item 13	0.578			
Item 10	0.577			
Item 19	0.564			
Item 7	0.558			
Item 4	0.534			
Item 18	0.466			
Item 16	0.466	-0.531		
Item 12			0.530	
Item 5	0.439			0.538

### Tests of normality and model structure

The results of the Kolmogorov-Smirnov and Shapiro Wilks tests (KS_test_ = 0.095, df =164, p < .001; SW_test_ = 0.096, df = 164, p = 0.004) indicated that the study phenomenon did not fit a normal distribution, and therefore any norms should not be based on a model of normality. The descriptive analysis of IEEC values yielded $\bar{x}$ = 182.35 (SD = 71.304, range 31–348). Tests of robustness, conducted in light of the skewed distribution, gave an approximation to the value of *Md* = 174 (Huber’s M-estimator = 175.91; Tukey’s biweight estimator = 173.28; Hampel’s M-estimator = 176.27; Andrews’ wave estimator = 173.21). The values indicate that our sample of critical care nurses had a moderate exposure to ethical conflict. The results are in line with previous studies [23,30], despite differences in the theoretical models and instruments used.

Moreover, regarding the organization of the elements that form the model of analysis of ethical conflict designed in this research, the results of the variance analysis for the variables “type of ethical conflict” and “IEEC” showed a statistically significant relation (p <0.05) between them for the 19 situations presented in ECNQ-CCV.

Finally, the inherent limitations of the research must be taken into account when interpreting the results. Firstly, it presents the intrinsic limitations associated to a self-administered questionnaire. Secondly, the analysed sample is made up of mainly women. Lastly, the fact that both the model for the analysis of ethical conflict and the design of a specific measuring instrument are new, represents a limitation when analysing criteria validity. It must be expected, however, that further studies in the context of critical care units will allow to perfect ECNQ-CCV and its use in other contexts will strengthen its discriminating value.

## Conclusions

The conceptual approach described in this paper makes a novel contribution to the study of ethical conflict, adopting as it does a perspective that goes beyond previous research in the field. Specifically, the proposed conceptual model of ethical conflict groups together different aspects or variables that, to date, have been treated either separately or incompletely by other authors. Thus, ethical conflict is considered here as the relationship between four variables: the frequency of occurrence of situations involving an ethical conflict, the degree of conflict produced, the individual’s exposure to ethical conflict and the type of ethical conflict experienced. This is the first study of ethical problems in nursing to consider the type of conflict experienced and to propose using an Index of Exposure to Ethical Conflict. The fact that there is a correlation between these two variables suggests that the types of conflict are associated with high or low levels of exposure to ethical conflict, as presented in the theoretical model developed in the research. This correlation must be analyzed in more detail in subsequent studies to determine the variability explained and effect size.

The Ethical Conflict in Nursing Questionnaire Critical Care Version (ECNQ-CCV), the instrument developed in order to operationalize the conceptual model, has been shown to have good psychometric properties, both in terms of reliability and construct validity. These results suggest that it is a suitable instrument for exploring ethical conflict among nurses working in critical care units.

The ECNQ-CCV is designed so as to enable both independent and combined studies of the four variables it evaluates: ‘frequency of concurrence’ , ‘degree of conflict’ , ‘exposure to ethical conflict’ and ‘type of ethical conflict’. This means that it can be used to analyse different factors that contribute to the phenomenon of ethical conflict, a line of research that would add more detailed knowledge about an enormously complex problem that affects all health professionals, especially nurses.

The present study represents the first evaluation of the ECNQ-CCV. However, the validation of measurement instruments is a complex and continuous process, and there is obviously a need for further studies that can administer the ECNQ-CCV to larger samples and in different types of critical care units. The results of these studies would enable both the instrument and the conceptual model on which it is based to be improved and made more robust. A further aim for the future would be to adapt the ECNQ-CCV to other clinical areas and other groups of health professionals.

## Abbreviations

CCU: Critical care unit; DEC: Degree of ethical conflict; DEFEC: Definition of ethical concepts; ECNQ-CCV: Ethical Conflict in Nursing Questionnaire-Critical Care Version; EFA: Exploratory factor analysis; FO: Frequency of occurrence of each scenario; IEEC: Index of exposure to ethical conflict; KStest: Kolmogorov-Smirnov Test; RI: Relevance of the item for exploing ethical conflict; SWtest: Shapiro Wilks test.

## Competing interests

The authors declare that they have no competing interests.

## Authors’ contributions

AFP and MTLL designed the study and prepared the manuscript. AFP administered the questionnaire and analysed the results. JGO provided statistical expertise and reviewed the statistical analysis of data. AFP, MTLL and JGO reviewed and revised the paper. All authors read and approved the final manuscript.

## Pre-publication history

The pre-publication history for this paper can be accessed here:

http://www.biomedcentral.com/1472-6939/14/22/prepub

## Supplementary Material

Additional file 1 Ethical Conflict Nursing Questionnaire - Critical Care Version (*) (**). Click here for file
