# Supplementary material for: Development process and initial validation of the Ethical Conflict in Nursing Questionnaire-Critical Care Version
Source: BMC Med Ethics. 2013 Jun 1;14:22. doi: 10.1186/1472-6939-14-22 (PMC3711987; doi:10.1186/1472-6939-14-22)
Supplement: Additional file 1 — Ethical Conflict Nursing Questionnaire - Critical Care Version (*) (**). [file 1472-6939-14-22-S1.docx]

**Additional File 1 - Ethical Conflict Nursing Questionnaire - Critical Care Version (*) (**).**

| Care nursing scenarios | A. How often have you found yourself in this situation? | B. Has this situation been an ethical problem for you? | C. Please highlight the option that best describes the moral state that you have experienced in this situation. |
| --- | --- | --- | --- |
| **Scenario 1**  Administering treatments and/or performing tests that I consider unnecessary because they serve merely to prolong a terminal, irreversible process. | *Never (Please do not answer B or C).* |  | Moral indifference, because I not feel involved in this situation. . |
|  | *Almost never.* | Not a problem at all. | Moral wellbeing, because my moral thought and action are clearly coherent with one another. |
|  | *At least once a year.* | Mildly problematic. | Moral uncertainty, because I’m not sure whether there is an ethical problem or not, or I recognize that there is a problem but is unclear about the ethical principles involved. |
|  | *At least once every six months.* | Fairly problematic. | Moral dilemma, because I must choose between two or more morally correct principles, each of which would lead to a distinct course of action. |
|  | *At least once a month.* | Considerably problematic. | Moral distress, because I recognize the ethical principles involved and I knows the right thing to do but is constrained by something or somebody from acting accordingly. |
|  | *At least once a week.* | Highly problematic. | Moral outrage, because I experience a sense of impotence in the face of an immoral action performed by others. |
| **Scenario 2**  Having to administer treatments and/or carry out procedures without the critical patient, who is conscious, knowing their purpose and the risks involved. | *Never (Please do not answer B or C).* |  | Moral indifference, because I not feel involved in this situation. . |
|  | *Almost never.* | Not a problem at all. | Moral wellbeing, because my moral thought and action are clearly coherent with one another. |
|  | *At least once a year.* | Mildly problematic. | Moral uncertainty, because I’m not sure whether there is an ethical problem or not, or I recognize that there is a problem but is unclear about the ethical principles involved. |
|  | *At least once every six months.* | Fairly problematic. | Moral dilemma, because I must choose between two or more morally correct principles, each of which would lead to a distinct course of action. |
|  | *At least once a month.* | Considerably problematic. | Moral distress, because I recognize the ethical principles involved and I knows the right thing to do but is constrained by something or somebody from acting accordingly. |
|  | *At least once a week.* | Highly problematic. | Moral outrage, because I experience a sense of impotence in the face of an immoral action performed by others. |
| **Scenario 3**  Caring for a patient who I believe should be on an ordinary hospital ward rather than in a critical care unit. | *Never (Please do not answer B or C).* |  | Moral indifference, because I not feel involved in this situation. . |
|  | *Almost never.* | Not a problem at all. | Moral wellbeing, because my moral thought and action are clearly coherent with one another. |
|  | *At least once a year.* | Mildly problematic. | Moral uncertainty, because I’m not sure whether there is an ethical problem or not, or I recognize that there is a problem but is unclear about the ethical principles involved. |
|  | *At least once every six months.* | Fairly problematic. | Moral dilemma, because I must choose between two or more morally correct principles, each of which would lead to a distinct course of action. |
|  | *At least once a month.* | Considerably problematic. | Moral distress, because I recognize the ethical principles involved and I knows the right thing to do but is constrained by something or somebody from acting accordingly. |
|  | *At least once a week.* | Highly problematic. | Moral outrage, because I experience a sense of impotence in the face of an immoral action performed by others. |
| **Scenario 4**  Carrying out interventions that put institutional or health service interests before those of the patient. | *Never (Please do not answer B or C).* |  | Moral indifference, because I not feel involved in this situation. . |
|  | *Almost never.* | Not a problem at all. | Moral wellbeing, because my moral thought and action are clearly coherent with one another. |
|  | *At least once a year.* | Mildly problematic. | Moral uncertainty, because I’m not sure whether there is an ethical problem or not, or I recognize that there is a problem but is unclear about the ethical principles involved. |
|  | *At least once every six months.* | Fairly problematic. | Moral dilemma, because I must choose between two or more morally correct principles, each of which would lead to a distinct course of action. |
|  | *At least once a month.* | Considerably problematic. | Moral distress, because I recognize the ethical principles involved and I knows the right thing to do but is constrained by something or somebody from acting accordingly. |
|  | *At least once a week.* | Highly problematic. | Moral outrage, because I experience a sense of impotence in the face of an immoral action performed by others. |
| **Scenario 5**  Failure to keep a patient’s clinical data confidential by sharing them with third parties or with people who are not directly involved in the patient’s care. | *Never (Please do not answer B or C).* |  | Moral indifference, because I not feel involved in this situation. . |
|  | *Almost never.* | Not a problem at all. | Moral wellbeing, because my moral thought and action are clearly coherent with one another. |
|  | *At least once a year.* | Mildly problematic. | Moral uncertainty, because I’m not sure whether there is an ethical problem or not, or I recognize that there is a problem but is unclear about the ethical principles involved. |
|  | *At least once every six months.* | Fairly problematic. | Moral dilemma, because I must choose between two or more morally correct principles, each of which would lead to a distinct course of action. |
|  | *At least once a month.* | Considerably problematic. | Moral distress, because I recognize the ethical principles involved and I knows the right thing to do but is constrained by something or somebody from acting accordingly. |
|  | *At least once a week.* | Highly problematic. | Moral outrage, because I experience a sense of impotence in the face of an immoral action performed by others. |
| **Scenario 6**  Administering treatments and/or carrying out interventions without the patient’s family knowing the objectives, benefits and risks involved (when the patient has consented to the family being informed) | *Never (Please do not answer B or C).* |  | Moral indifference, because I not feel involved in this situation. . |
|  | *Almost never.* | Not a problem at all. | Moral wellbeing, because my moral thought and action are clearly coherent with one another. |
|  | *At least once a year.* | Mildly problematic. | Moral uncertainty, because I’m not sure whether there is an ethical problem or not, or I recognize that there is a problem but is unclear about the ethical principles involved. |
|  | *At least once every six months.* | Fairly problematic. | Moral dilemma, because I must choose between two or more morally correct principles, each of which would lead to a distinct course of action. |
|  | *At least once a month.* | Considerably problematic. | Moral distress, because I recognize the ethical principles involved and I knows the right thing to do but is constrained by something or somebody from acting accordingly. |
|  | *At least once a week.* | Highly problematic. | Moral outrage, because I experience a sense of impotence in the face of an immoral action performed by others. |
| **Scenario 7**  Realizing that the analgesia and/or sedation being given to the patient is not effective enough and that the patient is suffering. | *Never (Please do not answer B or C).* |  | Moral indifference, because I not feel involved in this situation. . |
|  | *Almost never.* | Not a problem at all. | Moral wellbeing, because my moral thought and action are clearly coherent with one another. |
|  | *At least once a year.* | Mildly problematic. | Moral uncertainty, because I’m not sure whether there is an ethical problem or not, or I recognize that there is a problem but is unclear about the ethical principles involved. |
|  | *At least once every six months.* | Fairly problematic. | Moral dilemma, because I must choose between two or more morally correct principles, each of which would lead to a distinct course of action. |
|  | *At least once a month.* | Considerably problematic. | Moral distress, because I recognize the ethical principles involved and I knows the right thing to do but is constrained by something or somebody from acting accordingly. |
|  | *At least once a week.* | Highly problematic. | Moral outrage, because I experience a sense of impotence in the face of an immoral action performed by others. |
| **Scenario 8**  Using all available technical and/or human resources despite believing that they will produce no significant improvement in the clinical status of the critical care patient. | *Never (Please do not answer B or C).* |  | Moral indifference, because I not feel involved in this situation. . |
|  | *Almost never.* | Not a problem at all. | Moral wellbeing, because my moral thought and action are clearly coherent with one another. |
|  | *At least once a year.* | Mildly problematic. | Moral uncertainty, because I’m not sure whether there is an ethical problem or not, or I recognize that there is a problem but is unclear about the ethical principles involved. |
|  | *At least once every six months.* | Fairly problematic. | Moral dilemma, because I must choose between two or more morally correct principles, each of which would lead to a distinct course of action. |
|  | *At least once a month.* | Considerably problematic. | Moral distress, because I recognize the ethical principles involved and I knows the right thing to do but is constrained by something or somebody from acting accordingly. |
|  | *At least once a week.* | Highly problematic. | Moral outrage, because I experience a sense of impotence in the face of an immoral action performed by others. |
| **Scenario 9**  Working with medical staff who I consider to be professionally incompetent. | *Never (Please do not answer B or C).* |  | Moral indifference, because I not feel involved in this situation. . |
|  | *Almost never.* | Not a problem at all. | Moral wellbeing, because my moral thought and action are clearly coherent with one another. |
|  | *At least once a year.* | Mildly problematic. | Moral uncertainty, because I’m not sure whether there is an ethical problem or not, or I recognize that there is a problem but is unclear about the ethical principles involved. |
|  | *At least once every six months.* | Fairly problematic. | Moral dilemma, because I must choose between two or more morally correct principles, each of which would lead to a distinct course of action. |
|  | *At least once a month.* | Considerably problematic. | Moral distress, because I recognize the ethical principles involved and I knows the right thing to do but is constrained by something or somebody from acting accordingly. |
|  | *At least once a week.* | Highly problematic. | Moral outrage, because I experience a sense of impotence in the face of an immoral action performed by others. |
| **Scenario 10**  Administering treatments and/or carrying out interventions in accordance with the family’s wishes, despite knowing that these clash with the patient’s interests. | *Never (Please do not answer B or C).* |  | Moral indifference, because I not feel involved in this situation. . |
|  | *Almost never.* | Not a problem at all. | Moral wellbeing, because my moral thought and action are clearly coherent with one another. |
|  | *At least once a year.* | Mildly problematic. | Moral uncertainty, because I’m not sure whether there is an ethical problem or not, or I recognize that there is a problem but is unclear about the ethical principles involved. |
|  | *At least once every six months.* | Fairly problematic. | Moral dilemma, because I must choose between two or more morally correct principles, each of which would lead to a distinct course of action. |
|  | *At least once a month.* | Considerably problematic. | Moral distress, because I recognize the ethical principles involved and I knows the right thing to do but is constrained by something or somebody from acting accordingly. |
|  | *At least once a week.* | Highly problematic. | Moral outrage, because I experience a sense of impotence in the face of an immoral action performed by others. |
| **Scenario 11**  Administering treatments and/or carrying out procedures that are too aggressive given the status of the patient, and in so doing causing the patient additional suffering. | *Never (Please do not answer B or C).* |  | Moral indifference, because I not feel involved in this situation. . |
|  | *Almost never.* | Not a problem at all. | Moral wellbeing, because my moral thought and action are clearly coherent with one another. |
|  | *At least once a year.* | Mildly problematic. | Moral uncertainty, because I’m not sure whether there is an ethical problem or not, or I recognize that there is a problem but is unclear about the ethical principles involved. |
|  | *At least once every six months.* | Fairly problematic. | Moral dilemma, because I must choose between two or more morally correct principles, each of which would lead to a distinct course of action. |
|  | *At least once a month.* | Considerably problematic. | Moral distress, because I recognize the ethical principles involved and I knows the right thing to do but is constrained by something or somebody from acting accordingly. |
|  | *At least once a week.* | Highly problematic. | Moral outrage, because I experience a sense of impotence in the face of an immoral action performed by others. |
| **Scenario 12**  Working with a nurse or nursing assistant who I consider to be professionally incompetent. | *Never (Please do not answer B or C).* |  | Moral indifference, because I not feel involved in this situation. . |
|  | *Almost never.* | Not a problem at all. | Moral wellbeing, because my moral thought and action are clearly coherent with one another. |
|  | *At least once a year.* | Mildly problematic. | Moral uncertainty, because I’m not sure whether there is an ethical problem or not, or I recognize that there is a problem but is unclear about the ethical principles involved. |
|  | *At least once every six months.* | Fairly problematic. | Moral dilemma, because I must choose between two or more morally correct principles, each of which would lead to a distinct course of action. |
|  | *At least once a month.* | Considerably problematic. | Moral distress, because I recognize the ethical principles involved and I knows the right thing to do but is constrained by something or somebody from acting accordingly. |
|  | *At least once a week.* | Highly problematic. | Moral outrage, because I experience a sense of impotence in the face of an immoral action performed by others. |
| **Scenario 13**  Acting contrary to my own moral beliefs due to not having enough time to care properly for the patient. | *Never (Please do not answer B or C).* |  | Moral indifference, because I not feel involved in this situation. . |
|  | *Almost never.* | Not a problem at all. | Moral wellbeing, because my moral thought and action are clearly coherent with one another. |
|  | *At least once a year.* | Mildly problematic. | Moral uncertainty, because I’m not sure whether there is an ethical problem or not, or I recognize that there is a problem but is unclear about the ethical principles involved. |
|  | *At least once every six months.* | Fairly problematic. | Moral dilemma, because I must choose between two or more morally correct principles, each of which would lead to a distinct course of action. |
|  | *At least once a month.* | Considerably problematic. | Moral distress, because I recognize the ethical principles involved and I knows the right thing to do but is constrained by something or somebody from acting accordingly. |
|  | *At least once a week.* | Highly problematic. | Moral outrage, because I experience a sense of impotence in the face of an immoral action performed by others. |
| **Scenario 14**  Administering treatments in the context of a clinical trial or research project without, as a nurse, being given all the information I consider necessary to carry out this task. | *Never (Please do not answer B or C).* |  | Moral indifference, because I not feel involved in this situation. . |
|  | *Almost never.* | Not a problem at all. | Moral wellbeing, because my moral thought and action are clearly coherent with one another. |
|  | *At least once a year.* | Mildly problematic. | Moral uncertainty, because I’m not sure whether there is an ethical problem or not, or I recognize that there is a problem but is unclear about the ethical principles involved. |
|  | *At least once every six months.* | Fairly problematic. | Moral dilemma, because I must choose between two or more morally correct principles, each of which would lead to a distinct course of action. |
|  | *At least once a month.* | Considerably problematic. | Moral distress, because I recognize the ethical principles involved and I knows the right thing to do but is constrained by something or somebody from acting accordingly. |
|  | *At least once a week.* | Highly problematic. | Moral outrage, because I experience a sense of impotence in the face of an immoral action performed by others. |
| **Scenario 15**  Finding it difficult to give timely information to the patient and/or his/her family because the medical team discourages nurses from taking the initiative in this regard. | *Never (Please do not answer B or C).* |  | Moral indifference, because I not feel involved in this situation. . |
|  | *Almost never.* | Not a problem at all. | Moral wellbeing, because my moral thought and action are clearly coherent with one another. |
|  | *At least once a year.* | Mildly problematic. | Moral uncertainty, because I’m not sure whether there is an ethical problem or not, or I recognize that there is a problem but is unclear about the ethical principles involved. |
|  | *At least once every six months.* | Fairly problematic. | Moral dilemma, because I must choose between two or more morally correct principles, each of which would lead to a distinct course of action. |
|  | *At least once a month.* | Considerably problematic. | Moral distress, because I recognize the ethical principles involved and I knows the right thing to do but is constrained by something or somebody from acting accordingly. |
|  | *At least once a week.* | Highly problematic. | Moral outrage, because I experience a sense of impotence in the face of an immoral action performed by others. |
| **Scenario 16**  Caring for a patient without knowing whether or not he or she has made a living will declaration, or in the event that such a document exists not knowing its content. | *Never (Please do not answer B or C).* |  | Moral indifference, because I not feel involved in this situation. . |
|  | *Almost never.* | Not a problem at all. | Moral wellbeing, because my moral thought and action are clearly coherent with one another. |
|  | *At least once a year.* | Mildly problematic. | Moral uncertainty, because I’m not sure whether there is an ethical problem or not, or I recognize that there is a problem but is unclear about the ethical principles involved. |
|  | *At least once every six months.* | Fairly problematic. | Moral dilemma, because I must choose between two or more morally correct principles, each of which would lead to a distinct course of action. |
|  | *At least once a month.* | Considerably problematic. | Moral distress, because I recognize the ethical principles involved and I knows the right thing to do but is constrained by something or somebody from acting accordingly. |
|  | *At least once a week.* | Highly problematic. | Moral outrage, because I experience a sense of impotence in the face of an immoral action performed by others. |
| **Scenario 17**  Administering treatments and/or carrying out procedures without, as a nurse, having been previously involved in the decision to do so. | *Never (Please do not answer B or C).* |  | Moral indifference, because I not feel involved in this situation. . |
|  | *Almost never.* | Not a problem at all. | Moral wellbeing, because my moral thought and action are clearly coherent with one another. |
|  | *At least once a year.* | Mildly problematic. | Moral uncertainty, because I’m not sure whether there is an ethical problem or not, or I recognize that there is a problem but is unclear about the ethical principles involved. |
|  | *At least once every six months.* | Fairly problematic. | Moral dilemma, because I must choose between two or more morally correct principles, each of which would lead to a distinct course of action. |
|  | *At least once a month.* | Considerably problematic. | Moral distress, because I recognize the ethical principles involved and I knows the right thing to do but is constrained by something or somebody from acting accordingly. |
|  | *At least once a week.* | Highly problematic. | Moral outrage, because I experience a sense of impotence in the face of an immoral action performed by others. |
| **Scenario 18**  Failure to respect properly the privacy of the patient’s body when carrying out procedures and/or exploratory tests. | *Never (Please do not answer B or C).* |  | Moral indifference, because I not feel involved in this situation. . |
|  | *Almost never.* | Not a problem at all. | Moral wellbeing, because my moral thought and action are clearly coherent with one another. |
|  | *At least once a year.* | Mildly problematic. | Moral uncertainty, because I’m not sure whether there is an ethical problem or not, or I recognize that there is a problem but is unclear about the ethical principles involved. |
|  | *At least once every six months.* | Fairly problematic. | Moral dilemma, because I must choose between two or more morally correct |
|  |  |  | principles, each of which would lead to a distinct course of action. |
|  | *At least once a month.* | Considerably problematic. | Moral distress, because I recognize the ethical principles involved and I knows the right thing to do but is constrained by something or somebody from acting accordingly. |
|  | *At least once a week.* | Highly problematic. | Moral outrage, because I experience a sense of impotence in the face of an immoral action performed by others. |
| **Scenario 19**  Lacking the means (space) and/or resources (time) that would enable the clinical team to consider the ethical problems they have to face. | *Never (Please do not answer B or C).* |  | Moral indifference, because I not feel involved in this situation. . |
|  | *Almost never.* | Not a problem at all. | Moral wellbeing, because my moral thought and action are clearly coherent with one another. |
|  | *At least once a year.* | Mildly problematic. | Moral uncertainty, because I’m not sure whether there is an ethical problem or not, or I recognize that there is a problem but is unclear about the ethical principles involved. |
|  | *At least once every six months.* | Fairly problematic. | Moral dilemma, because I must choose between two or more morally correct principles, each of which would lead to a distinct course of action. |
|  | *At least once a month.* | Considerably problematic. | Moral distress, because I recognize the ethical principles involved and I knows the right thing to do but is constrained by something or somebody from acting accordingly. |
|  | *At least once a week.* | Highly problematic. | Moral outrage, because I experience a sense of impotence in the face of an immoral action performed by others. |

(*) The analysis of the psychometric properties of the ECNQ was carried out in relation to the original Spanish version of the instrument.

(**) You may request permission to author to use the questionnaire.

Types of ethical conflicts in ECNQ-CCV Relations between conflict areas and scenarios of ECNQ-CCV

Informed consent: Scenario 2, Scenario 6.

Confidentiality: Scenario 5.

Withholding and withdrawing treatments: Scenario 1.

Patient’s interests: Scenario 4, Scenario 10; Scenario 16.

Characteristics of an ethical environment: Scenario 19.

Procedures and treatments: Scenario 7, Scenario 11.

Interprofessional relationships: Scenario 9, Scenario 12, Scenario 15, Scenario 17.

Moral agency and professional values: Scenario 13.

Privacy: Scenario 18.

Research tasks: Scenario 14.

Resource management: Scenario 3, Scenario 8.

**Moral indifference** describes the stance of an individual who neither shows interest in nor takes a position on a matter of ethical concern.

**Moral wellbeing** describes to a positive state in which moral thought and action are clearly coherent with one another.

**Moral uncertainty** describes the states of an individual who either unsure whether there is an ethical problem or not, or recognizes that there is such a problem but is unclear about the ethical principles involved.

**Moral dilemma** arise when the individual must choose between two or more morally correct principles, each of which would lead to a distinct course of action.

**Moral distress** is felt when the individual recognizes the ethical principles involved and knows the right thing to do but is constrained by something or somebody from acting accordingly.

**Moral outrage** is a type of ethical conflict in which the individual experiences a sense of impotence in face of an immoral action performed by others.
